# Supplementary material for: Role of the Autism Risk Gene Shank3 in the Development of Atherosclerosis: Insights from Big Data and Mechanistic Analyses
Source: Cells. 2023 Oct 30;12(21):2546. doi: 10.3390/cells12212546 (PMC10647789; doi:10.3390/cells12212546)
Supplement: Supplementary file 1 [file cells-12-02546-s001.zip › cells-2626134-supplementary.pdf]

Table S1. Diagnostic codes for various diseases, treatments, or procedures

**Diseases**	**Diagnostic, treatment, or procedure codes**
Autism	299, 330.8, and F84
Hyperlipidemia	272, E71.30, E75.21, E75.22, E75.24, E75.3, E75.5, E75.6, E77, E78.0-E78.6, E78.70, E78.79, E78.8-E78.9, E88.1, E88.2, and E88.89
Myocardial infarction	410, I21, and I22
Ischemic stroke	433, 434, 436, I63, I65, I66, and I67.89
Antiplatelet therapy	B01AC
Computed tomography or magnetic resonance imaging	33084A, 33084B, 33085A, 33085B, 33067B, 33068B, 33069B, 33070B, 33071B, 33072B, 33098B, and 36021C
Cardiovascular death	433, 434, 436, 410, 414.00, 414.01, 414.02, 414.03, 414.04, 414.05, I21, I22, and I25

The codes corresponded to those specified in the *International Classification of Diseases*, *Ninth Revision*, *Clinical Modification*; *International Classification of Diseases*, *Tenth Revision*, *Clinical Modification*; or Anatomical Therapeutic Chemical classification.
